# Curcumin liposomes attenuate the expression of cigarette smoke extract-induced inflammatory markers IL-8 and IL-24 *in vitro*

**DOI:** 10.17179/excli2024-7467

**Published:** 2024-06-12

**Authors:** Vyoma K. Patel, Sofia Kokkinis, Gabriele De Rubis, Philip Michael Hansbro, Keshav Raj Paudel, Kamal Dua

**Affiliations:** 1 Discipline of Pharmacy, Graduate School of Health, University of Technology Sydney, Sydney, NSW 2007, Australia; 2 Faculty of Health, Australian Research Centre in Complementary and Integrative Medicine, University of Technology Sydney, Ultimo, Australia; 3 Faculty of Health and Medicine, School of Clinical Medicine, University of New South Wales, NSW 2052, Australia; 4 Pharmako Biotechnologies, Frenchs Forest, NSW 2086, Australia; 5 Centre for Inflammation, Centenary Institute and University of Technology Sydney, Faculty of Science, School of Life Sciences, Sydney 2007, Australia

## ⁯⁯⁯⁯⁯

Chronic obstructive pulmonary disease (COPD) represents the third leading cause of mortality that claims about 3.23 million deaths per year worldwide (Eapen et al., 2017[3]). It continues to be one of the major health concerns and is responsible for an increasing economic burden and morbidity (Vogelmeier et al., 2017[7]). COPD is a heterogenous disease that involves specific pathophysiological features including chronic inflammation, persistent airflow limitation, emphysema and altered lung function. It is primarily caused by prolonged exposure to cigarette smoke (CS), air pollutants, irritating gases, chemicals, and occupational dust (Yang et al., 2011[9]). Notably, CS is reported to induce the increased expression of inflammatory mediators such as IL (interleukin)-8, IL-24, tumor necrosis factor-alpha (TNF-α), nitric oxide, and granulocyte-monocyte colony stimulating factor (GM-CSF) from both alveolar macrophages and human broncho-epithelial cells (Paudel et al., 2022[6]). This release of inflammatory mediators plays a crucial role in causing progressive lung damage via impaired infiltration of inflammatory cells and thus, disease progression (Lugg et al., 2022[5]). Since COPD is irreversible, currently, the management of COPD is primarily dependent on managing symptoms and preventing progression of disease via the use of bronchodilators and corticosteroids. However, the clinical benefits of these agents are inimical to the patient as they are associated with numerous side effects, difficulty in using inhalation agents due to complex designs and associated cost. Despite advancement in therapeutic strategies in management of COPD however, to date, there is no cure. This highlights an urgent need for novel approaches that can cease the progression of this disease and reduce the associated mortality. In the quest for novel approaches, studies have shown that plant derived, or nutraceutical-based agents may aid controlling airway inflammation in respiratory diseases such as COPD. This is mainly due to these agents possessing potent effectiveness, fewer side effects, ease of usage, and affordability. 

Curcumin, derived from the **Curcuma longa *plant***, exhibits an intriguing array of potential health benefits as it has been shown to have antioxidant, anti-inflammatory, anti-viral, and anticancer properties (Kunnumakkara et al., 2017[4]). Studies have shown that curcumin has a therapeutic potential to treat a wide variety of inflammatory disorders including cardiovascular disease, osteoarthritis, and COPD (Kunnumakkara et al., 2017[4]). In COPD, curcumin has been shown to have a protective role in controlling the balance of various inflammatory and anti-inflammatory components. Notably, in COPD rat experimental model, curcumin ameliorated alveolar epithelial injury via reduction of the levels of pro-inflammatory cytokines such as IL-6, IL-8, IL-24, and tumor necrosis factor (TNF)-α (Xu and Liu, 2017[8]). Interestingly, one study has also shown that curcumin alleviated emphysema and aided to restore structural integrity of the alveolar epithelium in a COPD rat experimental model (Zhang et al., 2016[10]). Despite curcumin possessing various clinical benefits and potential to be a therapy in COPD, however, its poor water solubility and low bioavailability limits its clinical translation. To overcome this issue, several studies have shown encapsulation of nutraceuticals in advanced drug delivery systems as a beneficial strategy for effective delivery of curcumin to treat chronic inflammatory diseases due to an improvement in the molecule's stability, and the possibility to achieve controlled release (De Rubis et al., 2024[1]). Importantly, among the numerous signaling inflammatory molecules that promote COPD, IL-8 and IL-24 are exciting therapeutic targets, given they endorse numerous pathological hallmarks in COPD such as inducing pro-inflammatory, chemotactic, and matrix degradative responses into the airways. The overexpression and pathogenic role of IL-8 and IL-24 is not only highlighted in COPD but has also been shown in several inflammatory conditions such as allergic skin inflammation, rheumatoid arthritis (RA), psoriasis, lupus, and cardiovascular disease exacerbating further disease development (De Rubis et al., 2024[2]). Thus, the neutralization of IL-8 and IL-24 or blocking of the associated signaling pathways may lead to a resolution of these inflammatory conditions. In this context, in present study, we evaluated whether the curcumin-loaded liposomes (Curcumin-PlexoZome®) downregulate the expression levels of IL-8 and IL-24 *in vitro* in human BCi-NS1.1 bronchial epithelial cells exposed to 5 % cigarette smoke extract (CSE).

PlexoZome^® ^is a registered technology owned by Pharmako Biotechnologies, which utilizes phosphatidylcholine to encapsulate active ingredients within the lipid bilayer of a liposome, protecting it from degradation and increasing its stability. The liposomes were characterized by Pharmako Biotechnologies Pty Ltd through zeta analysis, particle size and polydispersity index (PDI). The sample of PlexoZome® curcumin used throughout this study consisted of a 0.1 % concentration of 95 % *Curcuma longa* extract. To conduct this study, minimally immortalized human bronchial epithelial cells (BCi-NS1.1) were grown using bronchial epithelial basal media with growth supplement as per manufacturer's instructions (Lonza). A research-grade reference cigarette 3R4F from Kentucky University, USA, was charred, and the smoke was bubbled in 10 mL of phosphate buffer solution (PBS). This was considered as a 100 % CSE solution which was filtered with a 0.22 μm filter and further diluted to 5 % CSE with fresh culture media. The cells were exposed to freshly prepared 5 % CSE within 30 minutes of preparation. The relative expression of IL-8 and IL-24 was determined using the Proteome Profiler Human XL Cytokine Array Kit (R&D Systems, Australia). The cells were seeded in 6 well plates and pre-treated with PlexoZome® Curcumin at an equivalent curcumin concentration of 5 µM for 1 hour, followed by 5 % CSE exposure. After treatment, cells were lysed in RIPA buffer (ThermoFisher Scientific, Australia) supplemented with protease inhibitors (Merck, Australia) and incubated on ice for 15 minutes to maximize protein extraction. Cell debris and membranes were removed through centrifugation at 14,000 g for 15 minutes at 4 °C. The protein content of the cleared supernatant was quantified with Pierce BCA Assay (ThermoFisher Scientific, Australia). A total of 300 µg proteins was loaded on each Proteome Profiler array membrane for the assessment of expression levels of IL-8 and IL-24, and the arrays were processed following manufacturer's instructions. The membranes were imaged using a ChemiDoc instrument (BioRad, Australia), and densitometric analysis for the protein expression quantitation was performed using ImageJ software. Data are presented as mean ± SEM and the statistical analyses were performed using one-way ANOVA followed by Tukey's multiple comparison test using PRISM GraphPad software (version 9.3). 

Firstly, untreated human bronchial epithelial cells (BCi-NS1.1 - control) express detectable levels of both IL-8 (supplementary information, Figure 1a) and IL-24 (supplementary information, Figure 1b). The relative expression of IL-8 and IL-24 was significantly upregulated by 5 % CSE exposure. 5 % CSE exposure resulted in a significant 5.2-fold increase in the expression of IL-8 (supplementary information, Figure 1a) and a significant 30.7-fold increase in the expression of IL-24 (supplementary information, Figure 1b). Treatment with PlexoZome® curcumin significantly reduced the levels of these two cytokines. PlexoZome® curcumin exerted a significant decrease of IL-8 levels by 70.6 % (supplementary information, Figure 1a) and significantly decreased the production of IL-24 by 92.7 % (supplementary information, Figure 1b) in comparison to the 5 % CSE alone group. The outcomes from this study revealed that Plexozome*®* curcumin at a concentration corresponding to 2.5 µM curcumin significantly downregulates the CSE-induced expression of IL-8 and IL-24 in BCi-NS1.1 human bronchial cells, showcasing the potential of this formulation as an effective therapy for COPD. 

Several studies have shown that the inhibition of the signaling pathways activated by IL-8 and IL-24 may prove as a viable strategy to inhibit the progression of COPD as these cytokines are involved in various pathological processes including infiltration of inflammatory factors, cellular chemotaxis, and production of matrix degradative factors into the airways. Most notably, previous studies have revealed that increased levels of IL-8 and IL-24 correlate with the severity of lung function impairment, inflammatory response, and development of lung epithelial lesions. Not only this but the increased levels and pathogenic role of IL-8 and IL-24 are also associated with progression of various other inflammatory conditions such as allergic skin inflammation, psoriasis, RA, lupus, cardiovascular disease, or infectious diseases (De Rubis et al., 2024[2]). These findings suggest that therapeutically targeting both IL-8 and IL-24 will not only aid in limiting the progression of COPD but can also prove as a potential therapy in the management of various other inflammatory conditions. 

Interestingly, in the quest of novel approaches, studies have revealed that curcumin, a major bioactive constituent found in phytomedicine turmeric, *Curcuma Longa* has a promising therapeutic potential in the treatment of various inflammatory disorders including COPD, cardiovascular disease, cancer, and osteoarthritis due to its anti-inflammatory, anti-viral, antioxidant, and anticancer properties. Despite curcumin possesses various clinical benefits and potential to be a therapy in COPD and other inflammatory disorders, however, its poor water solubility and low bioavailability outweighs its therapeutic benefits limiting its clinical translation. In this context, several studies have demonstrated the encapsulation of curcumin via liposomal technology to be one of the valuable approaches to deliver curcumin that is highly bioavailable with optimal therapeutic efficacy in the management of various inflammatory disorders. Importantly, our findings revealed the promising potential of Plexozome® curcumin as an effective therapy to improve the delivery of curcumin, showing that this formulation reduces the relative expression levels of inflammatory factors, IL-8, and IL-24 and thus, associated pathways that are activated by exposure to CS. Moreover, our results not only expand the findings of other studies that show curcumin-loaded liposomes to be an effective therapy to treat various inflammatory conditions but also have a greater impact in the treatment of COPD (De Rubis et al., 2024[1]). Considering the pivotal role of inflammatory factors such as IL-8 and IL-24 in promoting progression and development of COPD, the possibility that Plexozome® Curcumin acts as an anti-inflammatory and antioxidant agent warrants further functional and mechanistic investigation.

## Conflict of interest

This project has received funding from Pharmako Biotechnologies. The PlexoZome® Curcumin formulation we have tested is a product of Pharmako Biotechnologies, further information about it can be found on their official website: https://www.pharmako.com.au.

## Supplementary Material

Supplementary information
