# Supplementary material for: Curcumin liposomes attenuate the expression of cigarette smoke extract-induced inflammatory markers IL-8 and IL-24 in vitro
Source: EXCLI J. 2024 Jun 12;23:904–7. doi: 10.17179/excli2024-7467 (PMC11333702; doi:10.17179/excli2024-7467)
Supplement: Supplementary information [file EXCLI-23-904-s-001.pdf]

EXCLI Journal 2024;23:904-907 – ISSN 1611-2156
Received: June 04, 2024, accepted: June 11, 2024, published: June 12, 2024

Supplementary information to:

Letter to the editor:

CURCUMIN LIPOSOMES ATTENUATE THE EXPRESSION OF CIGARETTE SMOKE EXTRACT-INDUCED INFLAMMATORY MARKERS IL-8 AND IL-24 *IN VITRO*

Vyoma K. Patel[1,2,3], Sofia Kokkinis[1,2,4], Gabriele De Rubis[1,2], Philip Michael Hansbro[5], Keshav Raj Paudel[5], Kamal Dua[1,2,*]

1 Discipline of Pharmacy, Graduate School of Health, University of Technology Sydney, Sydney, NSW 2007, Australia
2 Faculty of Health, Australian Research Centre in Complementary and Integrative Medicine, University of Technology Sydney, Ultimo, Australia
3 Faculty of Health and Medicine, School of Clinical Medicine, University of New South Wales, NSW 2052, Australia
4 Pharmako Biotechnologies, Frenchs Forest, NSW 2086, Australia
5 Centre for Inflammation, Centenary Institute and University of Technology Sydney, Faculty of Science, School of Life Sciences, Sydney 2007, Australia

* **Corresponding author:** Dr. Kamal Dua, Discipline of Pharmacy, Graduate School of Health, University of Technology Sydney, Sydney, NSW 2007, Australia.
E-mail: kamal.dua@uts.edu.au

https://dx.doi.org/10.17179/excli2024-7467

This is an Open Access article distributed under the terms of the Creative Commons Attribution License (http://creativecommons.org/licenses/by/4.0/).

EXCLI Journal 2024;23:904-907 – ISSN 1611-2156
Received: June 04, 2024, accepted: June 11, 2024, published: June 12, 2024

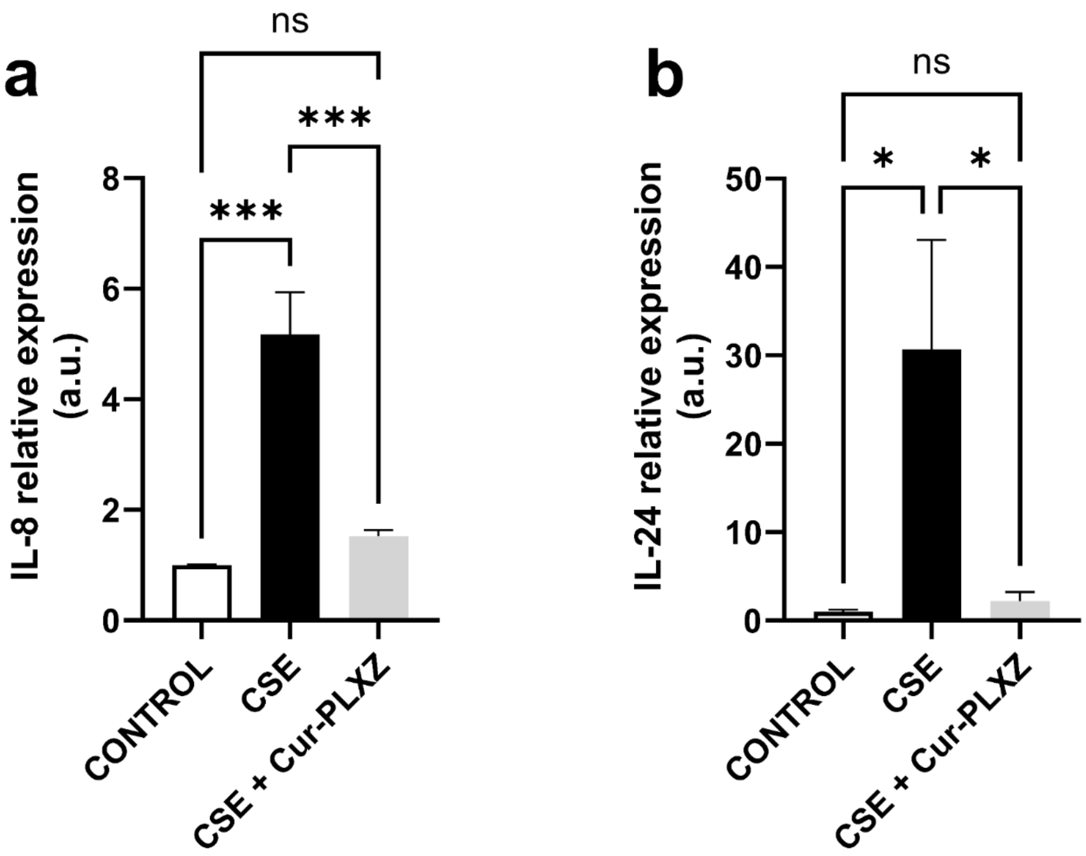

Figure 1: PlexoZome® curcumin counteracts the expression of IL-8 and IL-24 induced by CSE. BCi-NS1.1 cells were pre-treated with 2.5 µM PlexoZome® curcumin for one hour followed by 5 % CSE exposure for further 24 hours. The relative expression of IL-8 (**a**), and IL-24 (**b**), was measured using a Proteome Profiler Human XL Cytokine Array kit. Statistical analysis was conducted using PRISM GraphPad by one-way ANOVA followed by Tukey's multiple comparison test. Values are expressed as mean ± SEM (n=4). *: $p<0.05$; ***: $p<0.0001$; ns: not significant
